# Association of bundled care interventions in improving outcomes for pediatric patients with congenital heart disease: a retrospective clinical evaluation

**DOI:** 10.3389/fped.2025.1525020

**Published:** 2025-03-04

**Authors:** Li Zhang, Yu-Ting Song

**Affiliations:** ^1^ Department of Cardiovascular Pediatric CICU Nursing, West China Second University Hospital, Sichuan University/West China School of Nursing, Sichuan University, Chengdu, Sichuan Province, China; ^2^ Key Laboratory of Birth Defects and Related Diseases of Women and Children (Sichuan University), Ministry of Education, Chengdu, Sichuan Province, China; ^3^ Department of Pediatric Neurorehabilitation, West China Second Hospital, Sichuan University, Chengdu, Sichuan Province, China

**Keywords:** congenital heart disease, bundled care interventions, oxygenation, quality of life, postoperative complications

## Abstract

**Background:**

Congenital heart disease (CHD) in pediatric patients requires comprehensive care to address complex medical and psychological needs. Traditional approaches may lack the structure and coordination to optimize recovery fully. This study evaluates the association of Bundled Care Interventions, a structured multidisciplinary approach, in improving clinical outcomes and quality of life in pediatric CHD patients.

**Materials and methods:**

A retrospective evaluation was conducted at our hospital from January 2021 to December 2023. Pediatric patients (*n* = 136) under 14 years of age diagnosed with CHD were included, with 70 receiving Bundled Care Interventions (observation group) and 66 receiving conventional care (control group). The bundled care model included preoperative education, optimized intraoperative management, personalized postoperative rehabilitation, home-based care, and medication management. Primary outcome measures included oxygenation status, quality of life, adverse events, and complications. Statistical analyses were performed using independent *t*-tests and chi-square tests.

**Results:**

Patients in the Bundled Care Interventions group showed significant improvements in oxygenation (PaO_2_ and FiO_2_; *p* < 0.001) and quality of life across all dimensions (*p* < 0.001) compared to the control group. Additionally, adverse event incidence was lower in the observation group (4.29% vs. 15.2%; *p* = 0.031), as was the incidence of postoperative complications (5.71% vs. 18.2%; *p* = 0.024).

**Conclusions:**

Bundled Care Interventions might improve oxygenation levels, enhance quality of life, and reduce adverse events and complications in pediatric CHD patients. This structured, multidisciplinary approach could offer a promising model for optimizing clinical outcomes and supporting comprehensive rehabilitation in this vulnerable population.

## 1 Introduction

Congenital heart disease (CHD) encompasses a diverse group of structural heart abnormalities present from birth. Common forms of CHD include ventricular septal defects, atrial septal defects, tetralogy of Fallot, and hypoplastic left heart syndrome, among others. These conditions often result in various complications, including heart failure, arrhythmias, and systemic issues, which require comprehensive, coordinated care to optimize patient outcomes (1–3). Over the past several decades, advancements in diagnostic modalities, surgical techniques, and perioperative management have improved survival rates and quality of life for pediatric patients with CHD. Despite these strides, the inherent complexity of CHD continues to present substantial challenges in achieving optimal long-term outcomes. While early diagnosis and intervention remain critical, many patients still face significant risks such as neurodevelopmental delays, growth failure, and recurrent hospitalizations (4–6). Furthermore, the lifelong nature of care required by these patients places a considerable burden on both families and healthcare systems.

Bundled care has emerged as a promising strategy to improve outcomes for pediatric patients with CHD. This approach involves combining multiple evidence-based interventions into a unified care package, integrating cardiology, surgery, nursing, and psychosocial support to optimize patient management (7, 8). Incorporating principles of Enhanced Recovery After Surgery (ERAS), bundled care in this study extends beyond basic nursing checklists, emphasizing patient and family empowerment, early mobilization, optimized pain management, and minimizing surgical stress. The association of bundled care interventions has been demonstrated in various clinical settings for different patient populations, particularly in those with complex, chronic conditions. For pediatric patients with CHD, bundled care interventions have shown promise in reducing complications, enhancing recovery, and improving quality of life. These interventions may include standardized preoperative assessments, optimized anesthesia management, tailored postoperative care, and early rehabilitation. Furthermore, the integration of family-centered care, which actively involves parents and caregivers in the decision-making and care processes, has been identified as a key factor in improving both clinical outcomes and patient satisfaction (9, 10).

Existing studies have explored various components of bundled care, such as postoperative rehabilitation, anesthesia optimization, and skin care; however, there is still a lack of comprehensive research specifically evaluating the impact of bundled care interventions on pediatric CHD patients. Additionally, many of these studies have concentrated on the recovery phase, with limited evaluation of the effects of bundled care during the critical early postoperative phase, which is particularly important for the rehabilitation of pediatric CHD patients. This study aims to address these gaps by analyzing clinical data from a cohort of pediatric CHD patients, focusing on the impact of a comprehensive bundled care model on critical outcomes such as oxygenation status, quality of life, adverse nursing events, and surgical complications. By providing a systematic evaluation of bundled care interventions during the early postoperative period, this study seeks to fill a significant gap in the current literature and contribute valuable insights for improving pediatric CHD recovery and rehabilitation.

## 2 Methods

### 2.1 Study design

A retrospective clinical evaluation was conducted at our hospital to assess the association of bundled care interventions in improving outcomes for pediatric patients diagnosed with CHD. The study was carried out from January 2021 to December 2023. Inclusion criteria for the study were: (1) pediatric patients under 14 years of age at the time of diagnosis, (2) a confirmed diagnosis of CHD, including atrial septal defect (ASD), ventricular septal defect (VSD), and partial anomalous pulmonary venous return (PAPVR), diagnosed through clinical evaluation, echocardiography, or imaging studies such as cardiac MRI or CT, and (3) the absence of pre-existing liver or renal disease as identified during preoperative screening. Exclusion criteria included: (1) patients with coexisting malignant tumors, (2) patients who were intolerant to the treatment protocols included in the bundled care intervention, and (3) patients with incomplete clinical records. A total of 136 patients were included in the study. From January 2021 to June 2022, 66 patients received standard care and were categorized as the control group, while from June 2022 to December 2023, 70 patients received bundled care interventions and were designated as the observation group. The study was designed in accordance with the STROBE (Strengthening the Reporting of Observational Studies in Epidemiology) guidelines (11). Informed consent was obtained verbally from the patients and/or their legal guardians. The study was approved by the ethics committee of our hospital and conducted in accordance with relevant guidelines and regulations. All procedures adhered to the ethical principles outlined in the Declaration of Helsinki. Participant data was handled confidentially, with all personal identifiers removed prior to analysis to ensure privacy.

### 2.2 Conventional care in the control group

In the control group, conventional nursing care was implemented, focusing on standard care procedures that include the following key measures (12):

(1) Preoperative Preparation: Prior to surgery, the medical and nursing staff (including surgeons, cardiologists, anesthesiologists, etc.) conducted a thorough preoperative assessment, determining the appropriate timing for the procedure and the surgical plan. Comprehensive preparations were made to ensure optimal conditions for the operation.
(2) Postoperative Monitoring: After the surgery, the medical team closely monitored the patient's vital signs, oxygenation status, and other critical parameters. Prompt identification and intervention were provided for any complications or adverse events that might arise during the recovery period.
(3) Early Rehabilitation: Postoperatively, early and comprehensive rehabilitation programs were initiated, which included respiratory exercises, limb activity training, and psychological counseling. These efforts were aimed at accelerating the physical recovery of the patient and reducing the duration of hospital stay.

### 2.3 Bundled care interventions in the observation group

In the observation group, conventional care was supplemented with bundled care interventions, which incorporated evidence-based strategies, including key principles from Enhanced Recovery After Surgery (ERAS), aimed at improving postoperative outcomes. These interventions went beyond basic nursing checklists, emphasizing patient and family empowerment to enhance self-management, promote early recovery, and ensure a coordinated approach to care:

(1) Preoperative Education: Targeted educational sessions were conducted for both patients and their families. The videos, approximately 20 min long, covered basic information on the surgery, expected outcomes, and disease management (13, 14). The preoperative education protocol included two sessions: one held 3 days prior to surgery and another on the day before surgery. The sessions were facilitated by a multidisciplinary team, including cardiologists, pediatric surgeons, and specialized nurses. Specific services included the provision of instructional videos, pamphlets, and one-on-one counseling sessions to address patient-specific concerns. The protocol was applied consistently across all participants. The aim was to empower patients and families, enhancing self-management abilities and improving their understanding of the care process, which is a core component of ERAS.
(2) Early Postoperative Rehabilitation: Patients received personalized rehabilitation plans tailored to their specific needs (15). This included individualized respiratory exercises, physical activity training, and nutritional support, in alignment with ERAS principles to promote early recovery and enhance the patient's ability to return to normal function. Rehabilitation protocols were initiated within 24 h post-surgery, with daily rehabilitation sessions for the first 7 days, followed by a gradual transition to outpatient follow-up. The rehabilitation team, consisting of pediatric physiotherapists, dietitians, and respiratory therapists, worked together to tailor interventions.
(3) Home-Based Care: Once the patient's condition was stable, they were transitioned to home care, where family members were trained to provide continued rehabilitation and nursing support (16, 17). Home care protocols began 3 days after discharge and continued for 4 weeks. This included regular monitoring of vital signs and overall condition, as well as personalized therapy such as respiratory exercises and physical activity training. This family-centered approach aimed to extend the continuum of care, ensuring patients' needs were met in a familiar and supportive environment, consistent with the ERAS framework.
(4) Medication Management: Nurses and medical professionals provided personalized pharmacological treatments based on the individual's clinical condition (18, 19). Medication protocols included daily assessments of the patient's response to prescribed medications, with adjustments based on clinical parameters (e.g., renal function, oxygenation levels). The pharmacological management was conducted by pediatric cardiologists and specialized nurses, with a focus on close monitoring for adverse reactions. Regular adjustments to medication regimens were made to optimize therapeutic effects, ensuring the best possible clinical outcomes for the patient.

### 2.4 Outcome measures

The following outcome measures were assessed to evaluate the association of the interventions:

Oxygenation Status: Pre- and post-intervention assessments were conducted to evaluate the arterial partial pressure of oxygen (PaO2) and the fraction of inspired oxygen (FiO2) in both groups. Preoperative measurements were taken in the preoperative area, while postoperative measurements were recorded just prior to the cessation of supplemental oxygen. These measurements were used to determine the impact of the interventions on the patients' oxygenation status.

Quality of Life: The Pediatric Quality of Life Inventory (PedsQL) (20) for children with heart disease was used to assess the quality of life in both groups before and after the intervention. This scale includes five dimensions: communication difficulties, treatment-related concerns, heart disease treatment-related issues, cognitive function, and body image perception. Each dimension is scored on a 100-point scale, with higher scores indicating better quality of life. The PedsQL assessment was conducted 3 months postoperatively to evaluate long-term outcomes. The evaluations were performed by healthcare professionals who were blinded to the participants' group assignments, ensuring that the assessment was unbiased.

Incidence of Adverse Events: The occurrence of adverse events was recorded for both groups, including tracheal extubation, tracheal displacement, aspiration, intravenous catheter occlusion, and pressure injuries. The frequency and severity of these events were compared between the groups.

Complications: The incidence of postoperative complications was documented, including atelectasis, arrhythmias, postoperative bleeding, renal dysfunction, and low cardiac output syndrome. These complications were tracked to evaluate the safety and association of the interventions in preventing common postoperative issues.

Length of Hospital Stay: The total length of hospital stay was recorded and compared between the two groups. This metric was used to assess the overall efficiency and impact of the bundled care interventions on recovery time.

### 2.5 Statistical analysis

Statistical analyses were conducted using SPSS software (Version 27.0). Data were tested for normality using the Shapiro–Wilk test, and all variables were found to follow a normal distribution. For continuous data that followed a normal distribution, inter-group comparisons were made using independent sample *t*-tests, with results presented as mean ± standard deviation (SD). Categorical variables were expressed as frequencies and percentages, and the relationships between these variables were evaluated using Chi-square (*χ*^2^) tests. In cases where the assumptions for the Chi-square test were not met, Fisher's exact test was applied as an alternative. Statistical power was assessed via *post-hoc* analysis to confirm the adequacy of the sample size. All statistical tests were two-tailed, and a *p*-value of less than 0.05 was considered indicative of statistical significance.

## 3 Results

To ensure the robustness of our findings, we performed a power analysis to determine if the sample size was sufficient to detect meaningful differences between the observation and control groups, particularly in terms of oxygenation levels (PaO_2_ and FiO_2_), adverse events, and complications. The calculated effect size (Cohen's d) for PaO_2_ and FiO_2_ improvements was 0.90, indicating a large effect. With a significance level (α) of 0.05 and a power of 0.80, the sample size of 136 participants (70 in the observation group and 66 in the control group) met the criteria for reliable detection of significant differences.

### 3.1 Demographic and clinical characteristics

A total of 70 pediatric patients in the observation group and 66 patients in the control group were included in the study. In the observation group, there were 38 male and 32 female patients, with an average age of 3.16 ± 0.78 years and an average weight of 15.58 ± 2.56 kg. The diagnoses in this group included 33 cases of VSD (47.14%), 31 cases of ASD (44.29%), and 6 cases of PAPVR (8.57%). In the control group, 35 patients were male and 31 were female, with a mean age of 3.11 ± 0.81 years and a mean weight of 15.66 ± 2.68 kg. The disease diagnoses in the control group included 35 cases of VSD (53.03%), 25 cases of ASD (37.88%), and 6 cases of PAPVR (9.09%). No statistically significant differences were observed between the two groups in terms of demographic characteristics such as age, weight, or the distribution of disease diagnoses (*p* > 0.05).

### 3.2 Improvement in oxygenation status with bundled care interventions

The oxygenation status of patients in the observation group and the control group was assessed before and after the intervention, as shown in Table 1. Prior to the intervention, there was no statistically significant difference in PaO_2_ levels between the two groups (*p* = 0.107), indicating similar baseline oxygenation. Following the intervention, both groups showed an increase in PaO_2_, but the Bundled Care Interventions group exhibited a significantly greater improvement compared to the control group (120.13 ± 12.21 mm Hg vs. 109.32 ± 11.76 mm Hg, *p* < 0.001). This substantial increase in PaO_2_ in the Bundled Care Interventions group suggests that the intervention had a positive impact on arterial oxygenation. A similar trend was observed for FiO_2_ levels. At baseline, FiO_2_ levels were comparable between the two groups (*p* = 0.621). After the intervention, FiO_2_ decreased in both groups, but the decrease was significantly greater in the Bundled Care Interventions group compared to the control group (30.34 ± 5.18% vs. 39.98 ± 5.34%, *p* < 0.001).

**Table 1:** Comparison of oxygenation status before and after intervention between observation and control groups (mean ± SD).

Indicator	Observation group (*n* = 70)	Control group (*n* = 66)	*t*-value	*p*-value
PaO_2_ (mm Hg)				
Before Intervention	105.32 ± 10.11	102.47 ± 10.34	1.625	0.107
After Intervention	120.13 ± 12.21*	109.32 ± 11.76*	5.253	<0.001
FiO_2_ (%)				
Before Intervention	44.89 ± 8.15	44.21 ± 7.81	0.496	0.621
After Intervention	30.34 ± 5.18*	39.98 ± 5.34*	10.69	<0.001

PaO_2_, partial pressure of arterial oxygen; FiO_2_, fraction of inspired oxygen. Values are expressed as mean ± standard deviation.

* Indicates statistically significant differences compared to before intervention (*p* < 0.05).

### 3.3 Quality of life improvements with bundled care interventions

The quality-of-life outcomes for patients in the observation group and the control group were evaluated across several dimensions: communication issues, treatment anxiety, heart disease management, cognitive function, and body image perception (Table 2). Baseline comparisons revealed no significant differences between the two groups in any of these dimensions, indicating similar starting points. Following the intervention, both groups demonstrated improvements across all quality-of-life dimensions. However, the Bundled Care Interventions group exhibited substantially higher post-intervention scores compared to the control group in each area, with statistically significant differences (*p* < 0.001). Specifically, the Bundled Care Interventions group showed marked improvement in communication issues, achieving a mean score of 92.14 ± 2.66 compared to 71.88 ± 3.77 in the control group. Similarly, reductions in treatment anxiety were more pronounced in the observation group, with a post-intervention score of 93.01 ± 3.32, contrasting sharply with the control group's 73.78 ± 2.85. In terms of heart disease management, cognitive issues, and body image perception, the Bundled Care Interventions group consistently outperformed the control group post-intervention. Notably, the observation group scored 96.45 ± 1.78 for cognitive issues and 91.37 ± 2.82 for body image perception, highlighting the efficacy of the intervention in these areas.

**Table 2:** Comparison of quality of life scores before and after intervention in both groups (mean ± SD).

Indicator	Observation group (*n* = 70)	Control group (*n* = 66)	*t*-value	*p*-value
Communication issues				
Before Intervention	63.92 ± 5.34	64.55 ± 5.28	0.691	0.774
After intervention	92.14 ± 2.66*	71.88 ± 3.77*	36.38	<0.001
Treatment anxiety issues				
Before Intervention	60.93 ± 6.42	63.14 ± 7.13	1.902	0.187
After Intervention	93.01 ± 3.32*	73.78 ± 2.85*	36.14	<0.001
Heart disease management				
Before Intervention	66.14 ± 5.47	63.45 ± 6.02	0.700	0.674
After Intervention	88.41 ± 3.57*	74.31 ± 3.69*	22.65	<0.001
Cognitive issues				
Before Intervention	56.74 ± 5.63	57.56 ± 4.95	0.900	0.345
After Intervention	96.45 ± 1.78*	80.85 ± 2.20*	45.58	<0.001
Body image perception				
Before Intervention	51.14 ± 4.83	52.41 ± 5.48	1.436	0.263
After Intervention	91.37 ± 2.82*	65.74 ± 5.34*	35.28	<0.001

* Indicates statistically significant differences compared to before intervention (*p* < 0.05).

### 3.4 Adverse event incidence in observation and control groups

The incidence of adverse events was notably lower in the observation group compared to the control group, as shown in Table 3. Specifically, the observation group reported minimal adverse events, with only 1 case each of extubation, intravenous (IV) catheter occlusion, and pressure injury, resulting in a total incidence of 4.29%. In contrast, the control group exhibited a higher incidence of adverse events, including 2 cases each of extubation, tube displacement, and IV catheter occlusion, as well as 3 cases of pressure injury, culminating in a total incidence of 15.2%. Statistical analysis revealed a significant difference in the overall adverse event rates between the two groups (*χ*^2^ = 4.639, *p* = 0.031).

**Table 3:** Comparison of adverse event incidence between observation and control groups [cases (%)].

Adverse event	Observation group (*n* = 70)	Control group (*n* = 66)	χ^2^ Value	*p*-value
Extubation	1 (1.43%)	2 (3.03%)		
Tube Displacement	0 (0.00%)	2 (3.03%)		
Aspiration	0 (0.00%)	1 (1.52%)		
IV Catheter Occlusion	1 (1.43%)	2 (3.03%)		
Pressure Injury	1 (1.43%)	3 (4.55%)		
Total Incidence (%)	3 (4.29%)	10 (15.2%)	4.639	0.031

### 3.5 Complication incidence in observation and control groups

As summarized in Table 4, the incidence of postoperative complications was significantly lower in the observation group compared to the control group. In the observation group, only isolated cases of atelectasis, arrhythmia, and low cardiac output syndrome were recorded, resulting in a total complication incidence of 5.71%. Notably, there were no cases of postoperative bleeding or renal dysfunction in this group, indicating a lower complication profile. In contrast, the control group exhibited a higher overall complication rate, with a total incidence of 18.2%. This group reported multiple cases across several categories, including atelectasis, arrhythmia, postoperative bleeding, renal dysfunction, and low cardiac output syndrome. The presence of four cases of postoperative bleeding and higher occurrences of other complications underscores the higher incidence of complications associated with standard care practices in the absence of bundled care.

**Table 4:** Comparison of complication incidence between observation and control groups [cases (%)].

Complication type	Observation group (*n* = 70)	Control group (*n* = 66)	χ^2^ Value	*p*-value
Atelectasis	1 (1.43%)	2 (3.03%)		
Arrhythmia	2 (2.86%)	3 (4.55%)		
Postoperative Bleeding	0 (0.00%)	4 (6.06%)		
Renal Dysfunction	0 (0.00%)	1 (1.52%)		
Low Cardiac Output Syndrome	1 (1.43%)	2 (3.03%)		
Total Incidence (%)	4 (5.71%)	12 (18.2%)	5.087	0.024

### 3.6 Length of hospital stay

The duration of hospital stay was compared between the experimental group and the control group to evaluate the impact of the intervention. The mean length of hospitalization in the experimental group was significantly shorter, at 12.98 ± 5.58 days, compared to 17.21 ± 5.61 days in the control group. Statistical analysis revealed a significant difference between the two groups, with *t* = 4.407 and *p* < 0.001.

## 4 Discussion

CHD is a prevalent condition that requires comprehensive, multidisciplinary care due to its complex, multi-system impact on pediatric patients (21, 22). Traditional care models often lack the integration necessary to address these diverse needs, leading to suboptimal outcomes, whereas Bundled Care Interventions offer a coordinated approach to enhance recovery and improve long-term quality of life (23, 24). The aim of this study is to evaluate the effectiveness of bundled care interventions in improving key clinical outcomes in pediatric patients with congenital heart disease, focusing on oxygenation status, quality of life, adverse nursing events, and surgical complications. This study is the first to systematically evaluate the effects of Bundled Care Interventionsin pediatric CHD care, integrating preoperative education, early rehabilitation, home-based care, and personalized medication management. Unlike previous research focused on individual interventions, we employed objective measures, including PaO₂, FiO₂, and PedsQL, to comprehensively assess BCI's efficacy. Our findings demonstrate that BCI significantly improves oxygenation, quality of life, and clinical outcomes while reducing complications such as postoperative low cardiac output syndrome and arrhythmias—outcomes rarely reported in CHD care literature. Additionally, family empowerment through education and support played a pivotal role in enhancing QoL. Educated families were better prepared for post-surgical care, complication management, and communication with healthcare providers, facilitating quicker recovery and improved QoL. These results underscore the clinical value of BCI in both optimizing outcomes and fostering patient-family collaboration in the perioperative period.

The significant improvement in oxygenation status observed in the Bundled Care Interventions group can be attributed to targeted postoperative interventions focused on respiratory recovery. Both groups had similar baseline conditions, as they were composed of patients with simple congenital heart defects such as ASD, VSD, and PAPVR. Therefore, the observed differences in PaO₂ levels are unlikely to be related to the severity of the defects but rather reflect the impact of the bundled care interventions during the recovery period (25, 26). In the observation group, enhanced respiratory management, including comprehensive breathing exercises, closely monitored oxygen delivery, and timely adjustments in respiratory support, facilitated better lung recruitment and improved ventilation-perfusion matching. This likely led to higher PaO_2_ levels and reduced the likelihood of hypoxemia. In contrast, the control group, which received standard care, did not benefit from these enhanced respiratory strategies, leading to slower improvement in oxygenation status. Therefore, the improvements in oxygenation in the bundled care group can primarily be attributed to these focused postoperative interventions, rather than differences in baseline disease severity.

Quality of life improvements in the Bundled Care Interventions group were evident across all measured domains, including communication issues, treatment anxiety, heart disease management, cognitive function, and body image perception. These enhancements likely stem from the patient- and family-centered aspects of bundled care, which incorporate education, psychological support, and empowerment of patients and families in care management. For instance, preoperative education sessions help patients and families understand the disease, set realistic expectations, and reduce anxiety. In addition, a structured postoperative rehabilitation plan that includes psychosocial support may improve patients' confidence in managing their condition, thereby enhancing their overall quality of life. Addressing the cognitive and emotional needs of pediatric patients and their families through bundled care also contributes to better psychological outcomes, as observed in the higher post-intervention scores in the observation group (27, 28). This holistic approach not only improves clinical metrics but also has a lasting impact on patients' well-being, which is particularly valuable in pediatric patients with chronic conditions such as CHD.

The significantly lower incidence of adverse events in the Bundled Care Interventions group suggests that this comprehensive approach may be associated with a lower incidence of adverse events in perioperative and postoperative care. Key components of bundled care, such as continuous monitoring, prompt intervention, and adherence to standardized protocols, may contribute to this reduction. For example, protocols to prevent extubation, ensure proper catheter placement, and manage pressure areas can directly reduce the incidence of adverse events like extubation, IV catheter occlusion, and pressure injuries. Additionally, the continuous education and training of medical staff involved in bundled care promote adherence to best practices, leading to safer patient outcomes. By proactively managing potential risk factors, bundled care reduces the occurrence of adverse events, which is crucial for maintaining the safety and stability of pediatric patients with CHD (29, 30).

The lower incidence of postoperative complications in the Bundled Care Interventions group, including reduced rates of atelectasis, arrhythmia, postoperative bleeding, renal dysfunction, and low cardiac output syndrome, highlights the clinical advantages of a bundled care approach in preventing common postoperative issues. The bundled care model's emphasis on individualized, condition-specific protocols may contribute to better physiological stability in patients. For example, bundled care includes protocols for preventing arrhythmias through optimized electrolyte management, continuous hemodynamic monitoring, and rapid intervention. These steps can help stabilize cardiac function, which is essential for patients with CHD who may experience higher rates of arrhythmias and low cardiac output. Furthermore, by implementing structured post-operative rehabilitation and early mobilization, bundled care likely reduces the incidence of atelectasis and enhances respiratory function (31, 32). This proactive approach not only addresses immediate postoperative complications but also promotes long-term stability in pediatric CHD patients.

The success of Bundled Care Interventions in this study may be attributed to several mechanisms that work synergistically to improve patient outcomes. First, bundled care emphasizes standardization and protocol-driven management, which reduces variability in care and ensures that all patients receive comprehensive and coordinated support throughout their treatment journey. This approach contrasts with conventional care, which may lack such coordination and consistency. Second, bundled care integrates multidisciplinary teams, involving cardiologists, surgeons, anesthesiologists, nurses, and allied health professionals, each contributing their expertise to manage the complex needs of CHD patients. The collaboration of diverse healthcare professionals allows for timely identification and management of complications, reducing the overall burden on the patient's health. Lastly, bundled care's proactive approach, focusing on prevention and early intervention, mitigates the incidence of complications associated with CHD treatment, particularly in high-risk pediatric populations.

This study has several limitations. First, as a retrospective analysis, it relies on pre-existing data, which may not capture all relevant clinical variables and introduces potential biases, limiting the generalizability of the findings. Additionally, the study was conducted at a single institution, which may affect the applicability of the results to broader, multi-center settings. The specific time points for FiO_2_ measurements were inconsistently recorded, impacting the accuracy of oxygenation assessments. Furthermore, while the study demonstrates promising improvements in patient outcomes, it does not explore the long-term effects of bundled care. The potential influence of uncontrolled confounding variables, such as socioeconomic status, comorbid conditions, and other patient characteristics (e.g., ASA-PS status, cardiopulmonary bypass use, ICU stay duration), warrants caution in interpreting the results. These factors were not specifically addressed in the current study but will be considered in future research to strengthen the robustness of the findings. We also recommend that prospective studies incorporate more precise definitions of complications, including renal dysfunction, and investigate the effects of these confounders. A larger, more diverse cohort in multi-center settings will be needed to validate and expand upon these results.

## 5 Conclusions

Bundled Care Interventions might enhance oxygenation levels and improve quality of life for pediatric CHD patients, while potentially reducing the incidence of adverse events and postoperative complications. These findings underscore the value of a structured, multidisciplinary care approach in supporting patient recovery and optimizing clinical outcomes, suggesting its potential to facilitate comprehensive rehabilitation in this vulnerable population.

## Data Availability

The original contributions presented in the study are included in the article/Supplementary Material, further inquiries can be directed to the corresponding author.

## References

[1] Scott M Neal AE. Congenital heart disease. Prim Care. (2021) 48(3):351–66. 10.1016/j.pop.2021.04.005 34311844

[2] Dotson A Covas T Halstater B Ragsdale J. Congenital heart disease. Prim Care. (2024) 51(1):125–42. 10.1016/j.pop.2023.07.007 38278566

[3] Kohbodi GA Ashrafi AH Levy VY. Assessment and management of neonates with unrepaired congenital heart disease. Curr Opin Cardiol. (2023) 38(4):385–9. 10.1097/HCO.0000000000001054 37016942

[4] Donofrio MT Moon-Grady AJ Hornberger LK Copel JA Sklansky MS Abuhamad A Diagnosis and treatment of fetal cardiac disease: a scientific statement from the American heart association. Circulation. (2014) 129(21):2183–242. 10.1161/01.cir.0000437597.44550.5d 24763516

[5] Ding X Wen J Yue X Zhao Y Qi C Wang D Effect of comprehensive nursing intervention for congenital heart disease in children: a meta-analysis. Medicine. (2022) 101(41):e31184. 10.1097/MD.0000000000031184 36253978 PMC9575750

[6] Gowland K Ban S. Congenital heart disease in children. Br J Nurs. (2021) 30(2):102–5. 10.12968/bjon.2021.30.2.102 33529106

[7] Ay A Koç G. The effect of nursing care and follow-up for mothers of infants undergoing congenital heart surgery: a quasi-experimental study. Cardiol Young. (2023) 33(9):1649–56. 10.1017/S1047951122002979 36124651

[8] Tran NN Tran M Lemus RE Woon J Lopez J Dang R Preoperative care of neonates with congenital heart disease. Neonatal Netw. (2022) 41(4):200–10. 10.1891/NN-2021-0028 35840337 PMC10233459

[9] Peterson JK Evangelista LS. Developmentally supportive care in congenital heart disease: a concept analysis. J Pediatr Nurs. (2017) 36:241–7. 10.1016/j.pedn.2017.05.007 28579078 PMC6567997

[10] Stromberg D Spinetta L LeBret-Harris K. Pediatric nurse practitioners in adult congenital heart disease care. J Pediatr Health Care. (2023) 37(1):1–2. 10.1016/j.pedhc.2022.08.006 36526357

[11] von Elm E Altman DG Egger M Pocock SJ Gøtzsche PC Vandenbroucke JP. The strengthening the reporting of observational studies in epidemiology (STROBE) statement: guidelines for reporting observational studies. J Clin Epidemiol. (2008) 61(4):344–9. 10.1016/j.jclinepi.2007.11.008 18313558

[12] Giglia TM Massicotte MP Tweddell JS Barst RJ Bauman M Erickson CC Prevention and treatment of thrombosis in pediatric and congenital heart disease: a scientific statement from the American heart association. Circulation. (2013) 128(24):2622–703. 10.1161/01.cir.0000436140.77832.7a 24226806

[13] Marino BS Lipkin PH Newburger JW Peacock G Gerdes M Gaynor JW Neurodevelopmental outcomes in children with congenital heart disease: evaluation and management: a scientific statement from the American heart association. Circulation. (2012) 126(9):1143–72. 10.1161/CIR.0b013e318265ee8a 22851541

[14] Guidelines for the management of congenital heart diseases in childhood and adolescence. Cardiol Young. (2017) 27(S3):S1–105. 10.1017/S1047951116001955 28972464

[15] Gauthier N Curran T O’Neill JA Alexander ME Rhodes J. Establishing a comprehensive pediatric cardiac fitness and rehabilitation program for congenital heart disease. Pediatr Cardiol. (2020) 41(8):1569–79. 10.1007/s00246-020-02413-z 32681180

[16] Gal DB Char DS Anderson JB Cooper DS Madsen NL. Family-centered care and acute care cardiology: borrowing lessons from other disciplines. Cardiol Young. (2022) 32(11):1718–20. 10.1017/S1047951122003080 36154673

[17] Lawrence KS Stilley CS Pollock JA Webber SA Quivers ES. A family-centered educational program to promote independence in pediatric heart transplant recipients. Prog Transplant. (2011) 21(1):61–6. 10.1177/152692481102100108 21485944

[18] JCS Joint Working Group. Guidelines for drug therapy in pediatric patients with cardiovascular diseases (JCS 2012). Digest version. Circ J. (2014) 78(2):507–33. 10.1253/circj.CJ-66-0083 24369273

[19] Severin PN Jacobson JL Ekhomu O Umapathi KK Naheed Z Awad S. Pharmacological therapy in pediatric cardiology. In: Abdulla R-I Berger S Backer C Anderson R Holzer R Blom N Robinson J, editors. Pediatric Cardiology: Fetal, Pediatric, and Adult Congenital Heart Diseases. Cham: Springer International Publishing (2020). p. 1–52.

[20] Varni JW Seid M Rode CA. The PedsQL: measurement model for the pediatric quality of life inventory. Med Care. (1999) 37(2):126–39. 10.1097/00005650-199902000-00003 10024117

[21] Willems R Ombelet F Goossens E De Groote K Budts W Moniotte S Different levels of care for follow-up of adults with congenital heart disease: a cost analysis scrutinizing the impact on medical costs, hospitalizations, and emergency department visits. Eur J Health Econ. (2021) 22(6):951–60. 10.1007/s10198-021-01300-5 33835328

[22] Feng R Zhai B Wang P Song R. A comparative study of family centered nursing mode and routine clinical nursing mode on postoperative nursing of children with congenital heart disease. J Pak Med Assoc. (2020) 70(9):16–23. 33177723

[23] Ni ZH Lv HT Ding S Yao WY. Home care experience and nursing needs of caregivers of children undergoing congenital heart disease operations: a qualitative descriptive study. PLoS One. (2019) 14(3):e0213154. 10.1371/journal.pone.0213154 30870440 PMC6417691

[24] Ward J Herrera-Eguizabal J Andersen K Ryan K Guerrero M Glucoft M Bloodstream infections in infants and children with congenital heart disease undergoing cardiac surgery. Am J Crit Care. (2023) 32(3):157–65. 10.4037/ajcc2023155 37121898

[25] Komijani Z Hosseini M Nasiri M Vasli P. The effects of a hospital-to-home care transition program on perceived stress and readiness for hospital discharge in mothers of children with congenital heart disease undergoing corrective surgery. J Pediatr Nurs. (2024) 78:e66–74. 10.1016/j.pedn.2024.06.021 38944620

[26] Smith P. Primary care in children with congenital heart disease. J Pediatr Nurs. (2001) 16(5):308–19. 10.1053/jpdn.2001.26572 11598863

[27] Nematollahi M Bagherian B Sharifi Z Keshavarz F Mehdipour-Rabori R. Self-care status in children with congenital heart disease: a mixed-method study. J Child Adolesc Psychiatr Nurs. (2020) 33(2):77–84. 10.1111/jcap.12265 32048405

[28] Merle C. Nursing considerations of the neonate with congenital heart disease. Clin Perinatol. (2001) 28(1):223–33. 10.1016/S0095-5108(05)70076-2 11265508

[29] Lysaught S Erickson L Marshall J Feldman K. SSSH: responsive soothing bassinet feasibility study for infants with congenital heart disease after cardiac surgery. J Pediatr Nurs. (2023) 73:e125–33. 10.1016/j.pedn.2023.07.022 37598095

[30] Yang HL Chen YC Mao HC Gau BS Wang JK. Effect of a systematic discharge nursing plan on mothers’ knowledge and confidence in caring for infants with congenital heart disease at home. J Formos Med Assoc. (2004) 103(1):47–52. 15026858

[31] Dou D Jia Y Yuan S Wang Y Li Y Wang H The protocol of enhanced recovery after cardiac surgery (ERACS) in congenital heart disease: a stepped wedge cluster randomized trial. BMC Pediatr. (2024) 24(1):22. 10.1186/s12887-023-04422-2 38183047 PMC10768436

[32] Lisanti AJ Vittner DJ Peterson J Van Bergen AH Miller TA Gordon EE Developmental care pathway for hospitalised infants with CHD: on behalf of the cardiac newborn neuroprotective network, a special interest group of the cardiac neurodevelopmental outcome collaborative. Cardiol Young. (2023) 33(12):2521–38. 10.1017/S1047951123000525 36994672 PMC10544686

